# Functionality of the *Paracoccidioides* Mating α-Pheromone-Receptor System

**DOI:** 10.1371/journal.pone.0047033

**Published:** 2012-10-04

**Authors:** Jéssica A. Gomes-Rezende, Ana G. Gomes-Alves, João F. Menino, Marco A. Coelho, Paula Ludovico, Paula Gonçalves, Mark H. J. Sturme, Fernando Rodrigues

**Affiliations:** 1 Life and Health Sciences Research Institute (ICVS), School of Health Sciences, University of Minho, Braga, Portugal; 2 ICVS/3B’s - PT Government Associate Laboratory, Braga/Guimarães, Portugal; 3 Centro de Recursos Microbiológicos (CREM), Departamento de Ciências da Vida, Faculdade de Ciências e Tecnologia, Universidade Nova de Lisboa, Caparica, Portugal; Universidade de Sao Paulo, Brazil

## Abstract

Recent evidence suggests that *Paracoccidioides* species have the potential to undergo sexual reproduction, although no sexual cycle has been identified either in nature or under laboratory conditions. In the present work we detected low expression levels of the heterothallic *MAT* loci genes *MAT1-1* and *MAT1-2*, the α-pheromone (*PBα*) gene, and the α- and **a**-pheromone receptor (*PREB* and *PREA*) genes in yeast and mycelia forms of several *Paracoccidioides* isolates. None of the genes were expressed in a mating type dependent manner. Stimulation of *P. brasiliensis MAT1-2* strains with the synthetic α-pheromone peptide failed to elicit transcriptional activation of *MAT1-2, PREB* or *STE12*, suggesting that the strains tested are insensitive to α-pheromone. In order to further evaluate the biological functionality of the pair α-pheromone and its receptor, we took advantage of the heterologous expression of these *Paracoccidioides* genes in the corresponding *S. cerevisiae* null mutants. We show that *S. cerevisiae* strains heterologously expressing *PREB* respond to Pbα pheromone either isolated from *Paracoccidioides* culture supernatants or in its synthetic form, both by shmoo formation and by growth and cell cycle arrests. This allowed us to conclude that *Paracoccidioides* species secrete an active α-pheromone into the culture medium that is able to activate its cognate receptor. Moreover, expression of *PREB* or *PBα* in the corresponding null mutants of *S. cerevisiae* restored mating in these non-fertile strains. Taken together, our data demonstrate pheromone signaling activation by the *Paracoccidioides* α-pheromone through its receptor in this yeast model, which provides novel evidence for the existence of a functional mating signaling system in *Paracoccidioides*.

## Introduction

*Paracoccidioides* species are thermodimorphic ascomycete fungi that occur in a non-pathogenic mycelial form at environmental temperatures (below 25°C), and switch to a pathogenic multiple-budding yeast-form at the mammalian host temperature (37°C). This fungus is the etiological agent of paracoccidioidomycosis (PCM), a systemic mycosis that is prevalent in Latin America and has high incidence in Colombia, Venezuela and Brazil [1], [2]. Host infection occurs through inhalation of infective airborne conidia (asexual spores) or mycelial propagula from the environment that differentiates into the pathogenic yeast form in the lungs and may disseminate to other organs, thereby producing a disseminated mycosis [3].

While many studies have addressed key virulence factors (reviewed in [4]) and the cell cycle of *P. brasiliensis* [5], several other basic aspects of its biology still remain to be elucidated, in particular its ecology and whether or not it is capable of sexual reproduction. Sexual structures have not been observed for this fungus and it is therefore thought to only reproduce asexually. This is in contrast to the phylogenetically closely related dimorphic fungus *Histoplasma capsulatum*, which exhibits a clear heterothallic sexual reproduction mode [6], [7]. The absence of a sexual cycle has been noted for over 20% of fungal species [8], which is surprising considering the proposed advantages of a sexual cycle over solely asexual reproduction [9], [10]. Nonetheless, in the last decade several studies have uncovered extant sexual cycles in several pathogenic fungi that were previously considered to be “asexual” (viz. *Aspergillus fumigatus, Candida albicans* and *Candida tropicalis*) [11]–[14]. *C. albicans* was shown to undergo mating *in vitro* as well as *in vivo* in a mammalian host [11], [12]. The identification of sexual reproduction in *A. fumigatus* required lengthy incubations of mating pairs under particular conditions [13], though a recent study identified some highly fertile *A. fumigatus* strains, that were capable of completing the sexual cycle more rapidly [15]. Moreover, a study by Pyrzak *et al* [16] showed that low transcriptional activity of mating-type regulators might be the cause of the low fertility observed in *A. fumigatus*. These studies suggest that the discovery of sexual reproduction in asexual fungi may require specific culturing conditions, and show as well that fungi may have regulatory constraints that hinder expression of the mating system.

**Figure 1:**
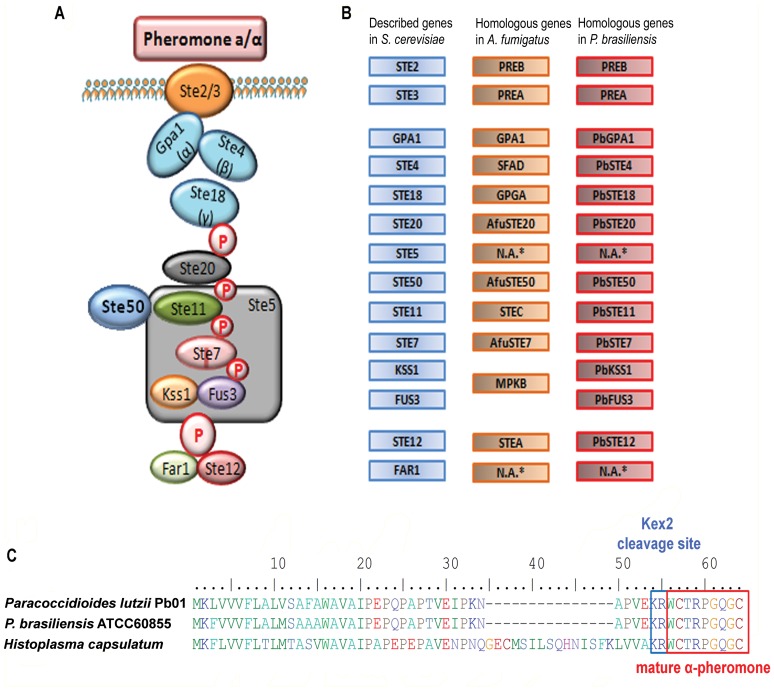
The genome of *Paracoccidioides* encodes an α-pheromone. (A) Mating pheromone signaling pathway described in *S. cerevisiae* [21]. (B) *S. cerevisiae* mating pheromone signaling pathway components and the homologous genes annotated in *Paracoccidioides* and *A. fumigatus* databases. Genes not annotated in databases are denoted N.A. (C) Alignment of α-pheromone precursor peptide sequences from *Paracoccidioides* strains and *H. capsulatum*. The Kex2 peptidase recognition site (KR) and the mature pheromone are indicated by blue and red boxes, respectively.

Even though sexual reproduction has not yet been observed in *Paracoccidioides*, there are indications that it may occur. Firstly, phylogenetic and population genetics studies of *Paracoccidioides* species brought to light the existence of four different phylogenetic lineages, namely: S1 (species 1 from Brazil, Venezuela, Peru, Paraguay and Argentina), PS2 (phylogenetic species from Brazil and Venezuela), PS3 (phylogenetic species from Colombia), and the “Pb01-like” group, the latter of which has been proposed to constitute a new species named *Paracoccidioides lutzii* [17], [18]. Genealogical analysis of isolates from these lineages presented evidence for genetic recombination within and between lineages of the *Paracoccidioides* genus [17], indicative of sexual reproduction. Additional support for the existence of a sexual cycle came from the identification of two idiomorphic mating-type genes (*MAT1-1* and *MAT1-2*) in *Paracoccidioides* isolates, exhibiting high similarities to mating-type genes of other filamentous ascomycetes [19], [20]. A 1∶1 distribution of the *MAT* loci was observed among the isolates consistent with a heterothallic sexual reproduction mode, and gene expression of *MAT* genes was confirmed. Torres *et al*. also tested *in vitro* mating of opposite mating types of *Paracoccidioides* under different conditions, but were unable to confirm the production of true sexual structures [20]. This could mean that the production of functional sexual structures requires additional stimuli and/or different environmental conditions or that *Paracoccidioides* mating mechanisms are not functioning efficiently at a genetic or biochemical level. Besides the identification of two *MAT* idiomorphs [19], [20], *MAT1-1* comprising an α-domain gene and *MAT1-2* containing an HMG gene, whole genome sequencing of three *Paracoccidioides* isolates further revealed the presence of most components of the mating-pheromone mitogen-activated protein kinase (MAPK) signaling pathway and meiosis specific genes described in other fungi [21], [22]. The MAPK pathway involved in sexual reproduction is well described in *S. cerevisiae* and conserved among fungi (Figure 1A and B). This pathway is activated when the mating pheromone binds to the respective membrane receptor, leading to the dissociation of the Gα subunit of the G-protein coupled to the membrane receptor. The cascade is composed of Ste20 (protein kinase), Ste11 (MAP kinase kinase kinase), Ste7 (MAP kinase kinase) and Fus3 and Kss1 (MAP kinases). These kinases are supported on a scaffold provided by the Ste5 protein. The signal from this cascade is transferred to Far1 (cyclin-dependent kinase inhibitor) and Ste12 (transcription factor) leading to transcription of mating-related genes [21]. *Paracoccidioides* harbors all the components of the cascade found in *S. cerevisiae*, except for the Ste5 and Far1 homologs [22] (Figure 1B). In this work, we identified a non-annotated gene encoding a mating α-pheromone (*PBα*) and evaluated its expression in *Paracoccidioides*, as well as the expression of its cognate α-pheromone receptor (PreB). As the observation of mating in *Paracoccidioides* remains elusive, despite the apparent availability of the entire genetic makeup required for pheromone signaling, associated with the fact that genetic tools for functional studies in this fungus are limited [23], [24], we set out to determine whether or not components of the mating-pheromone signaling would be able to function in the heterologous host *S. cerevisiae*.

Although mating gene expression could not be induced in the tested *P. brasiliensis MAT1-2* strains stimulated with synthetic Pbα pheromone, we were able to provide evidence for the functionality of the Pbα pheromone and its receptor PreB in the heterologous expression host *S. cerevisiae*. Our study provides a framework for further studies into the functionality of mating components present in *Paracoccidioides* aiming to identify potential restrictions at the molecular level that reduce mating fertility in this pathogenic fungus.

## Materials and Methods

### Strains and Culture Conditions

*Paracoccidioides* and *S. cerevisiae* strains used in this study are listed in Table 1. *S. cerevisiae* deletion strains were obtained from the EUROSCARF deletion strain collection. For maintenance, *S. cerevisiae* strains were grown at 30°C on YEPD solid medium (0.5% yeast extract, 1% peptone, 2% glucose and 2% agar) or YNB minimal medium dropout plates (6.7% Yeast Nitrogen Base without amino acids, 2% glucose and 2% agar), supplemented to meet auxotrophic requirements. For experimental procedures, *S. cerevisiae* strains were grown in YEPD or YNB broth at 26°C and 150 rpm. *Paracoccidioides* yeast strains were maintained at 37°C by periodic subculturing on brain heart infusion (BHI) solid medium supplemented with 1% glucose (1.6% agar). For assays the yeast form was grown in BHI broth supplemented with 1% glucose at 37°C and 200 rpm. The mycelium form was grown in synthetic McVeigh Morton (MMcM) medium [25] at 24°C, 150 rpm. *Escherichia coli* strains were grown in Lysogeny broth (LB) medium (1% tryptone, 0.5% yeast extract, 1% NaCl) at 37°C, 220 rpm, and supplemented with selective antibiotics where appropriate.

**Table 1:** Strains used in this study.

Strain	Genotype^a^	Source
*S. cerevisiae*		
BY4741	*MAT* **a** (*his3Δ1 leu2Δ0 met15Δ0 ura3Δ0)*	EUROSCARF
BY4742	*MAT*α (*his3Δ1 leu2Δ0 lys2Δ0 ura3Δ0)*	EUROSCARF
BY4741 *STE2*Δ	*MAT* **a** *STE2*::kanMX4	EUROSCARF
BY4742 *MF(α)1*Δ	*MAT*α *MF(α)*1::kanMX4	EUROSCARF
BY4742 *MF(α)2*Δ	*MAT*α *MF(α)*2::kanMX4	EUROSCARF
AGΔScα	*MAT*α *MF(α)1*::kanMX4 *MF(α)2*::hph	This study
AGLPbα	*MAT*α *MF(α)1*::kanMX4 *MF(α)2*::hph pLPbα	This study
AGMPbα	*MAT*α *MF(α)1*::kanMX4 *MF(α)2*::hph pMPbα	This study
AGLPreB	*MAT*a *STE2*::kanMX4 pLPreB	This study
*Paracoccidioides* isolates		
*P. lutzii* Pb01	*MAT1-1* (Pb01)^b^	Chronic PCM/Goiás - Brazil [18]
*P. brasiliensis* T8B1	*MAT1-1* (S1)^b^	Armadillo/Botucatu - Brazil [17]
*P. brasiliensis* Pb03	*MAT1-2* (PS2)^b^	Chronic PCM/São Paulo - Brazil [44]
*P. brasiliensis* ATCC60855	*MAT1-2* (PS3)^b^	Sputum/Colombia [45]

a –Plasmids listed in Table 2.

b –Phylogenetic species as defined by Matute et al. [17] and Teixeira et al. [18].

### Construction of a *MF(α)1/2* Double Mutant in *S. cerevisiae*

For heterologous expression of the *Paracoccidioides* α-pheromone (*Pbα*) in *S. cerevisiae,* a double mutant for both *S. cerevisiae* α-pheromones *MF(α)1* and *MF(α)2* genes was constructed. To obtain a double mutant with two different antibiotic resistance cassettes replacing the *MF(α)1* and *MF(α)2* genes, we first replaced the geneticin cassette (*KanMx*) in *S. cerevisiae* strain BY4742 Δ*MF(α)2* by a hygromycin B cassette (*hph*). To this end plasmid pAG34, carrying the *hph* gene under the control of a TEF promoter and terminator, was used. This plasmid allows for homologous recombination of the *hph* cassette with the *KanMX* cassette in EUROSCARF deletion strains, as this cassette carries the same TEF promoter and terminator. Plasmid pAG34 was digested with restriction enzyme XhoI (FastDigest, Fermentas), and subsequently transformed in strain BY4742 Δ*MF(α)2* using the lithium acetate method as described [26]. Transformants were isolated on medium with hygromycin as a selective marker. Replacement of the *KanMX* cassette in gene *MF(α)2* by a *hph* cassette was confirmed by PCR. Subsequently, the strain BY4742 Δ*MF(α)1* was transformed with the *hph* cassette with *MF(α)2* flanking regions (amplified using primers MFα2-HR-HPH-Fw and MFα2-HR-HPH-Rev, and Phusion DNA Polymerase (Finnzymes)). Double mutants for the *MF(α)1/2* genes of *S. cerevisiae* were isolated on YEPD selective plates (supplemented with hygromycin and geneticin) and correct genomic insertion was confirmed by PCR. PCR reactions were performed with primers described in Table S1, using the DyNAzyme II DNA polymerase kit (Finnzymes) and genomic DNA from transformants as a template.

### Construction of Heterologous Expression Plasmids

All vectors reported in this study are listed in Table 2. For heterologous expression of *Paracoccidioides* mating-related genes the tetracycline-repressed expression vectors pCM189 (low-copy) and pCM190 (high-copy) were used, both containing the uracil marker (*URA3*). *P. brasiliensis* α-pheromone (GenBank accession number JX429928) and *PREB* genes were amplified from Pb01 mycelium-specific cDNA, using proof-reading Phusion DNA Polymerase (Finnzymes). The amplified PCR products included short terminal sequences homologous to the vector cloning site, to allow for cloning by homologous recombination (primers - Table S1). PCR products and linearized vectors were transformed in the respective *S. cerevisiae* deletion strain BY4741 Δ*STE2* (for *PREB*) or AGΔScα (for *Paracoccidioides* α-pheromone). *PREB* cloned in the low-copy vector pCM189 was designated pLPreB, while *Paracoccidioides* α-pheromone cloned in both low and multi-copy vectors, were designated pLPbα and pMPbα, respectively. Transformants were isolated on YNB uracil dropout medium and confirmed by PCR. PCR reactions were performed with primers described in Table S1, using the DyNAzyme II DNA polymerase kit (Finnzymes) and genomic DNA from transformants as a template.

**Table 2:** Plasmids used in this study.

Plasmid	Construction	Resistance/auxotrophic marker	Source
pAG34	–	Ampicillin, Hygromycin B	EUROSCARF
pLPbα	pCM189::PBα	Ampicillin/Uracil	This study
pMPbα	pCM190::PBα	Ampicillin/Uracil	This study
pLPreB	pCM189::PREB	Ampicillin/Uracil	This study

### Identification of *Paracoccidioides* Mating-related Genes

Identification of *Paracoccidioides* mating-related genes was performed using BLAST searches against a genome database of isolates Pb01, Pb03 and Pb18, available at the Broad Institute (http://www.broadinstitute.org/). In order to identify the α-pheromone gene we performed TBLASTN searches against *Paracoccidioides* transcripts and genomic sequences (E-value cut-off 1e-1) using the precursor protein sequence of the *Histoplasma capsulatum* α-pheromone PPG1 as a query (GenBank: ACU27365.1). Subsequently, transcript hits were translated in all six reading frames using ORF Finder, to identify the position and reading frame of the pheromone sequence. The **a** and α pheromone receptor genes (*PREA* and *PREB*, respectively) are annotated by the Broad Institute and the genes involved in the intracellular signaling pathway were obtained after performing a BLASTN using the known sequences of other ascomycete fungi. Pheromone receptor topology was predicted using TMHMM 2.0 and visualized with TMRPres2D.

To confirm the *in silico* identified sequences for the *Paracoccidioides* α-pheromone and PreB, their respective transcript sequences were amplified from cDNA (primers - Table S1) using a combination of DyNAzyme II and Phusion DNA polymerases (Finnzymes). The PCR products were cloned with the TOPO TA Cloning Kit for sequencing (Invitrogen) following the manufacturer’s protocol. Plasmid was extracted from *E. coli* strain DH5α using the QIAprep Spin Miniprep Kit (Qiagen) and sequenced at STAB VIDA (Portugal). Alpha-pheromone transcript sequences have been deposited at GenBank under accession numbers JX429928 and JX429929.

### Growth Arrest Assays

For growth arrest assays the mature *Paracoccidioides* α-pheromone (Pbα – WCTRPGQGC) was synthesized at Metabion (Germany) and synthetic *S. cerevisiae* α-pheromone (MFα - WHWLQLKPGQPMY) was obtained from GenScript (USA). Halo assays to monitor pheromone-induced growth arrest were performed to establish the biological activity of Pbα in a *S. cerevisiae STE2* deletion strain expressing the *Paracoccidioides* α-pheromone receptor PreB (AGLPreB). The strains *S. cerevisiae* BY4741 and AGLPreB were grown for 16 hrs in YNB minimal drop-out medium (supplemented with the appropriate amino acids) to an optical density at 640 nm (OD_640_) of 1.0 to 1.4. Yeast cells at 10^6^ and 5×10^6^ cells/ml were embedded in soft agar (0.8%), spread on YEPD plates and dried. Subsequently, 10 µl of synthetic Pbα or MFα (at 2 mg/mL), and 10 µl of Pbα pheromone extracted from *P. brasiliensis* cultures and from AGLPbα was spotted on plates and incubated for 16–24 h at 30°C. Halo development was monitored and halo areas measured. Each experiment was repeated four times.

Cell growth arrest upon pheromone stimulation was also assessed by measuring Colony Forming Units (CFUs) at different time points along incubation with synthetic pheromones. Cultures at an initial concentration of 3.3×10^7^ cells/ml were incubated with Pbα (4 and 40 µg/ml) for strain AGLPreB or MFα (2 and 20 µg/ml) for strain BY4741. At indicated time-points samples were taken and serial dilutions plated on the respective selective media. CFU values were determined after 48 hours of incubation at 30°C. Growth rate constants (µ) were determined from the respective CFU growth curves.

### Shmoo Assay

Different strains of *S. cerevisiae* were grown in YNB minimal drop-out medium (supplemented with the appropriate amino acids) to an optical density at 640 nm (OD_640_) of 1.0 to 1.4. Cells were resuspended in YEPD to 4×10^6^ cells/mL and incubated at 26°C with either Pbα (at 4 and 40 µg/mL) or MFα (at 2 and 20 µg/mL), respectively. The percentage of cell-shmooing was determined at different times and cell images were taken on a Zeiss Axioskop equipped with a Carl Zeiss AxioCam (Carl Zeiss, Jena). Shmoo counts were done for 200–300 cells, and each experiment was repeated five times.

### α-pheromone Isolation

α-pheromone from Pb01/Pb03 and Pb01/ATCC60855 mycelium co-cultures, Pb01, ATCC60855, Pb03 and T8B1 mono-culture or AGLPbα culture was extracted using the resin Amberlite® XAD®2, according to the protocol described elsewhere [27]. In brief, resin was washed in distilled water and conditioned for α-factor isolation in methylene chloride-methanol (1∶3 v/v). Pheromone isolation from *Paracoccidioides* mycelium co-cultures was performed by direct addition of resin to the co-cultures and incubation of co-cultures for 14 days at 24°C, 150 rpm. For Pbα pheromone extraction from AGLPbα the resin was directly added to a stationary phase culture and incubated for 2 days at 30°C, 200 rpm. After incubations, resin was washed several times with distilled water to remove mycelium or yeast cells and α-pheromone subsequently extracted by incubation in 40% methanol for 2 hrs at 40°C. Methanol extracts were dried using SpeedVac lyophilisation and resuspended in sterile distilled water.

### Cell Cycle Analysis by Flow Cytometry

Strains BY4741 and AGLPreB were grown in YNB minimal drop-out medium YNB (supplemented with the appropriate amino acids) to an optical density at 640nm (OD_640_) of 1.0–1.4. Cells were resuspended in YEPD to 4×10^6^ cells/mL and incubated at 26°C with either Pbα or MFα at 40 or 20 µg/mL, respectively. Cell cycle analysis was performed for different time points. Cell treatment was adapted from Fortuna et al. [28]. Briefly, cells were fixed by resuspending in 500 µl 70% ethanol, and subsequently centrifuged and washed with 1 mL of sodium citrate buffer (50 mM pH 7.5). Cells were resuspended in 850 µl of sodium citrate buffer and 125 µl of RNase A (2 mg/mL in Tris-EDTA pH 8.0) and incubated at 50°C for 1 h. After the addition of 50 µl of proteinase K (20 mg/mL) samples were incubated at 50°C for 1 h. Cells were transferred to a cytometry tube and stained overnight at 4°C with SYBR Green I (final concentration ranging 0.01X to 100X in Tris-EDTA pH 8.0). Triton X-100 (0.25% v/v in 50 mM sodium citrate buffer pH 7.5) was added and the sample was sonicated with three consecutive ultrasound pulses at 40W for 2 sec with an interval of 2 sec between each pulse. Cell cycle analysis was performed by flow cytometry (FCM) on a BD™ LSR II flow cytometer. A minimum of 50,000 cells per sample were acquired at low/medium flow rate. Offline data were analyzed with the flow cytometry analysis software package FlowJo 7.6.1. Each experiment was repeated four times.

### Quantitative Mating Assays

Quantitative mating assays were performed based on the protocol by Guthrie and Fink [29]. Cells were grown in YNB minimal drop-out medium (supplemented with the appropriate amino acids) to exponential phase. Cells were mixed on a 0.45 µm pore membrane (Amersham Hybond-N nylon membranes, GE Healthcare), using a vacuum filtration system (TPP), at different concentrations (1× corresponds to 1.5×10^7^ cells). Membranes were placed on the surface of YEPD plates and incubated at 30°C for 5 hrs. For mating assays with the addition of the synthetic pheromone, cells were not filtrated, but incubated in YEPD media for 5 hrs. Cells were resuspended in sterile water and plated on YNB drop-out plates without methionine and lysine to select for diploids, and on plates without methionine or lysine in order to select for haploid cells. Mating efficiency was calculated as [30]:
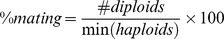



### Real-time-PCR Gene Expression Analysis and *Pbα* Pheromone Induction

For basal expression analysis yeast cultures were inoculated from a single colony and grown to exponential phase, by incubation for 7 days at 37°C, 200 rpm, during which medium was refreshed once after 4 days. Mycelium cultures were obtained by incubating these yeast cultures for 7 days at 24°C, 150 rpm, to allow for complete yeast-to-mycelium conversion. Total RNA was extracted from *Paracoccidioides* yeast and mycelial cells using Trizol (Invitrogen) standard method for cellular disruption, complemented with heat shock treatment (45 min at 65°C followed by 60 min at −80°C) and bead-beating using 0.1 mm silica/zirconium beads. Total RNA was treated with DNase I (Ambion) and DNA-free total RNA (1 µg) reverse transcribed using the DyNAmo™ cDNA Synthesis Kit (Finnzymes). cDNA samples were used as templates in order to measure the basal level expression of the *MAT1-1*, *MAT1-2*, *PREA*, *PREB, GPA1, STE4, STE18, STE20, STE50, STE11, STE7, KSS1, STE12* and α-pheromone genes in yeast and mycelium. Quantitative RT-PCR (qRT-PCR) was performed on the CFX96 Real-Time PCR Detection System (Bio-Rad), and qRT-PCR amplification performed using the *Sso* Fast EvaGreen Supermix kit (Bio-Rad), according to the manufacturer’s protocol. For all samples DNA contamination was discarded, as the utilization of the isolated RNA as a template gave no amplification in qRT-PCR. The thermal cycling conditions comprised: initial denaturation at 95°C for 30 sec, 40 cycles of denaturation at 95°C for 5 sec and primer annealing and elongation for 5 sec at appropriate temperature. After PCR cycling, melt-curves were obtained from 65–95°C at 0.5°C increments.

For pheromone induction experiments, *Paracoccidioides* strains were grown as mycelium to exponential phase in MMcM medium [25] and synthetic Pbα pheromone was added to 50 µg/mL. Samples were taken at intervals, and RNA extraction and real-time PCR were performed as described above. All measurements were performed in triplicate and relative expression levels determined using the ΔC_T_ method [31] versus β-tubulin (*TUB2*) as a reference gene [32]. The primers used are listed in Table S1.

### Statistical Analysis

Data are reported as the mean ± standard error of the mean of at least four independent repetitions of each assay. The statistical analyses were performed using the SPSS program version 19 and GraphPad software.

## Results

### The *Paracoccidioides sp.* Genome Encodes a Mating α-pheromone

The genome of *Paracoccidioides* harbors most of the mating- and meiosis-specific genes, with the exception of *S. cerevisiae STE5* and *FAR1* homologues [22] (Figure 1). Interestingly, while the genus appears to be heterothallic, based on the presence of either the *MAT1-1* or *MAT1-2* idiomorph in all strains examined, all three fully sequenced isolates (Pb01, Pb03 and Pb18) encode both the receptors for the mating **a-**pheromone (PreA) and the α-pheromone (PreB). The corresponding mating pheromone genes were however not annotated. To this end we used the α-pheromone precursor sequence of the phylogenetically closely related species *Histoplasma capsulatum* (PPG1: GenBank ACU27365.1) as a query to perform a TBLASTN database search in all six reading frames against transcripts and genomic sequences of *P. brasiliensis*/*P. lutzii*. Results showed an ORF encoding an α-pheromone homologue in *P. lutzii* Pb01 and *P. brasiliensis* Pb03 and Pb18 (data not shown). To evaluate the biological significance of this *in silico* predicted pheromone gene we confirmed the sequence of cDNA generated from *P. lutzii* Pb01 and *P. brasiliensis* ATCC60855 mycelium cultures (Figure S1). Sequence analysis of the genomic DNA and the cDNA revealed that the pheromone gene appears to be interrupted by an intron of 79 bp (Figure S1), where exon-intron boundaries obey the canonical GT-AG rule [33].

The alignment between the *Histoplasma* and *Paracoccidioides* precursor pheromone protein sequences (Figure 1C) showed highly conserved regions along the entire sequence (53% identity), and both contain a Kex2 protease (prototypical eukaryotic prohormone-processing enzyme) recognition sequence (KR). Cleavage at this site would release the mature pheromone sequence (WCTRPGQGC), which shows an identity of 100% with *H. capsulatum* and 77% with *A. fumigatus*, respectively, and contains residues that are conserved among other fungal α-pheromones in general [34]. The predicted small ORF encoding the α-pheromone (designated *PBα*) overlaps with the annotated hypothetical genes PAAG_00855, PABG_01384 and PADG_03979 of Pb01, Pb03 and Pb18 respectively, and for Pb01 the *PBα* gene is located on the opposite DNA strand of PAAG_00855.

### Mating-related Gene Expression in *Paracoccidioides sp.* Yeast and Mycelium

**Figure 2:**
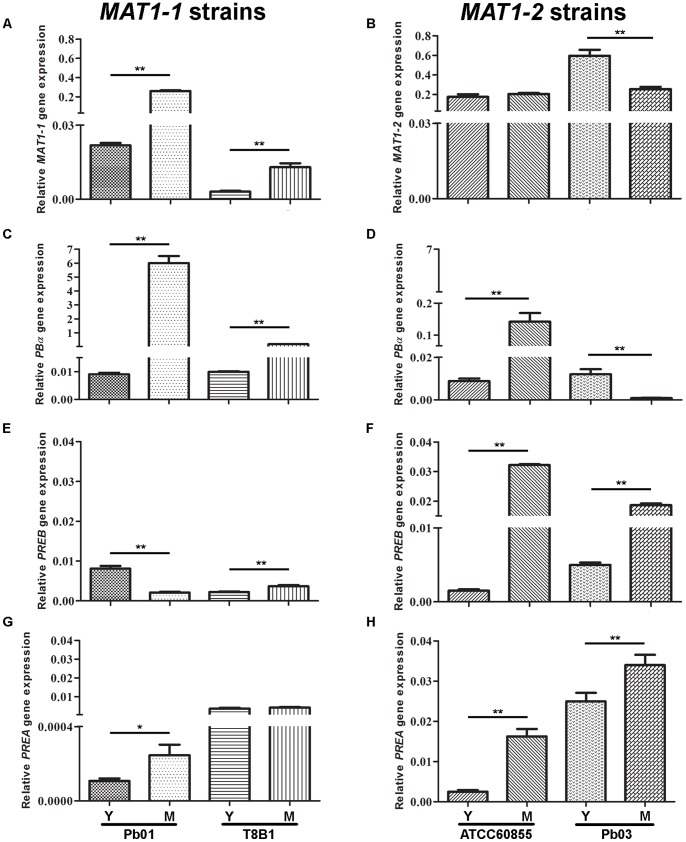
*Paracoccidioides* expresses the α-pheromone and the a- and α-receptor genes independently of the mating type. Mating gene expression of *Paracoccidioides MAT1-1* strains (Pb01 and T8B1) and *MAT1-2* strains (ATCC60855 and Pb03) in yeast (Y) and mycelial (M) form. Expression levels of *MAT1-1* (A) and *MAT1-2* (B), *PBα* (B, C), *PREB* (E, F) and *PREA* (G, H) genes were measured. Error bars are indicated, and asterisks show significant differences (*p<0.05 or **p<0.01).

As described above, *Paracoccidioides* harbors most genes implicated in mating, but it has not been demonstrated that this fungus undergoes a sexual cycle. Therefore we set out to assess if *Paracoccidioides* isolates produce mating-specific transcripts. To this end, expression levels of the *in silico* identified mating-related genes were monitored by qRT-PCR in yeast and mycelial cultures of strains representing both mating types (*MAT1-1* and *MAT1-2*), and including isolates for each of the four phylogenetic groups previously described (Figure 2) [17], [18]. Expression of *Paracoccidioides MAT1-1* or *MAT1-2* genes under basal conditions was shown to occur at low levels in both yeast and mycelium (Figure 2). For the strains encoding the *MAT1-1* idiomorph, expression of the α-pheromone and the **a**-pheromone receptor *PREA* was expected, whereas for *MAT1-2* strains **a**-pheromone and the α-pheromone receptor *PREB* expression was envisaged. However, instead we observed low expression levels of both receptors in strains of both mating-types. The *MAT1-1* strains expressed both pheromone receptors at low levels but there is a clear difference between yeast and mycelial forms (Figure 2E, G). Although overall expression is low, the *MAT1-2* strains show significantly higher expression levels of both receptors (*PREA* and *PREB*) in the mycelium when compared with the yeast form (Figure 2F, H). Moreover, the *MAT1-2* strains in mycelial form have higher expression levels of both receptors when compared to the *MAT1-1* strains. Previous studies have shown that some heterothallic ascomycetes express pheromone receptors in a mating-type independent manner, as is the case for *A. fumigatus*, *Neurospora crassa* and *Candida glabrata* [35]–[37]. Similarly, the α-pheromone is expressed by strains of both mating-types, although with higher values for the *MAT1-1* strains (Figure 2C, D). In addition, the expression levels the α-pheromone are significantly higher in the mycelium form, with the exception of Pb03 (Figure 2C, D). However, expression levels are very low for all strains, except for Pb01 that shows a high level of expression in mycelium form.

### The α-pheromone does not Elicit Mating Response in *Paracoccidioides*

**Figure 3:**
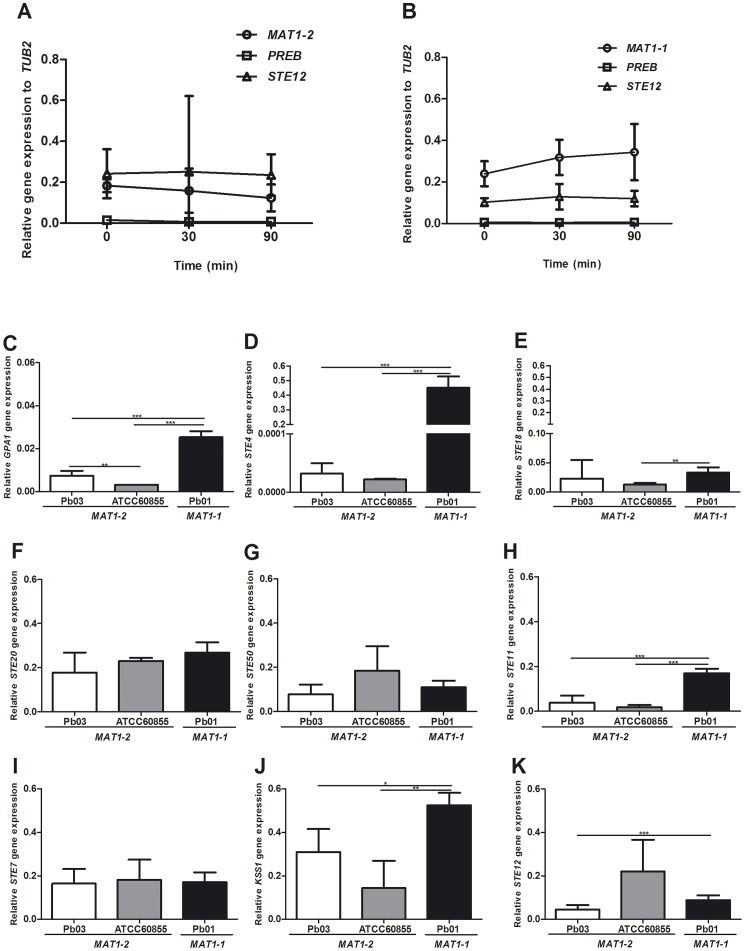
α-pheromone does not induce mating gene expression in *Paracoccidioides*. Mating gene expression at different time points in *P. brasiliensis* ATCC60855 (A) and *P. lutzii* Pb01 (B) in mycelium form, upon induction with 50 µg/mL of synthetic Pbα pheromone. Basal expression levels of MAPK signaling pathway components in the mycelium form of *P. brasiliensis* Pb03 and ATCC60855 (*MAT1-2*) and *P. lutzii* Pb01 (*MAT1-1*) (C-K). Error bars are indicated, and asterisks show significant differences (*p<0.05, **p<0.01, ***p<0.001).

To assess the biological activity of the identified α-pheromone of *Paracoccidioides sp.* we tested the ability of a synthetic peptide identical to the predicted mature pheromone to trigger a transcriptional response of mating-related genes in *P. brasiliensis MAT1-2* strains ATCC60855 and Pb03 in mycelium form. Our data show that expression of *MAT1-2*, *PREB* and *STE12* genes did not increase upon pheromone stimulation in *P. brasiliensis* ATCC60855 (Figure 3A) and Pb03 (data not shown). Taking into consideration that *MAT1-1* strains also express *PREB*, we further evaluate the transcriptional response of *P. lutzii* Pb01 to the α-pheromone. Our results show that, as for the *MAT1-2* strains, the pheromone did not trigger a transcriptional response of the *MAT*-related genes analysed (Figure 3B). The non-response of *Paracoccidioides* species to α-pheromone led us to question whether or not the molecular players involved in the signalling cascade were being expressed under basal condition in mycelium form. Our data shows that all the components (Figure 1 A) are expressed at low levels (Figure 3 C–K). Moreover, comparative analysis of the expression levels (Figure 2 and 3) highlight that in particular the upper components of the signalling cascade (*PREB*, *GPA1*, *STE4* and *STE18*) show the lowest expression levels, which could explain the inability of *Paracoccidioides* to respond to the α-pheromone.

To further evaluate the functionality of the pair α-pheromone and its cognate receptor PreB and considering the observed low mating gene expression and the inability to induce pheromone signaling in *Paracoccidioides,* we continued our studies using the well established *S. cerevisiae* system as a heterologous model.

### *Paracoccidioides* α-pheromone Promotes *shmoo* Formation in *S. cerevisiae* Expressing α-pheromone Receptor

To assess the functionality of the predicted *Paracoccidioides* α-pheromone in the heterologous model *S. cerevisiae*, the *PREB* gene, encoding the α-pheromone receptor, was cloned into a constitutive expression vector and was used to transform a *S. cerevisiae ste2* null mutant. The resulting strain is hereafter referred to as AGLPreB.

**Figure 4:**
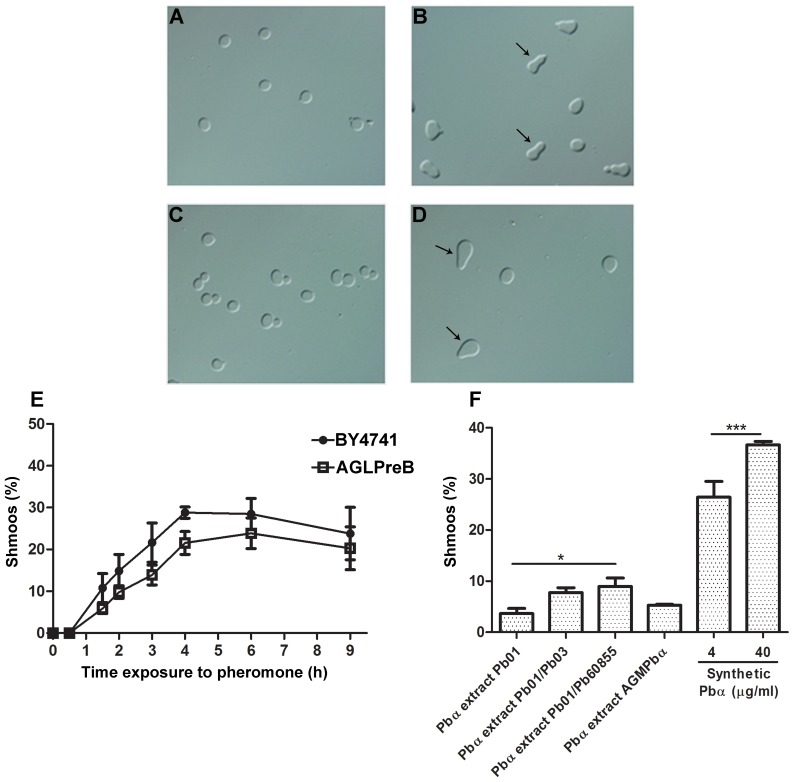
α-pheromone activates PreB receptor and induced *shmoo* formation in the *S. cerevisiae* model. *Shmoo* formation in *S. cerevisiae* BY4741 induced with 20 µg/ml synthetic MFα pheromone after 0 and 5 hrs induction (A and B), and *S. cerevisiae* AGLPreB induced with 40 µg/ml synthetic Pbα pheromone after 0 and 5 hrs induction (C and D). Quantification of *shmoo* formation using *S. cerevisiae* BY4741 stimulated with MFα (2 µg/mL) or *S. cerevisiae* AGLPreB stimulated with Pbα (4 µg/mL) (E). Induction of *S. cerevisiae* AGLPreB using Pbα pheromone extracted from *P. lutzii* Pb01 monoculture or *Paracoccidioides* co-cultures (Pb01/Pb03 and Pb01/Pb60855), Pbα pheromone extracted from AGMPbα culture, as well stimulation with the synthetic Pbα pheromone (40 µg/mL) (F). Error bars are indicated, and asterisks show significant differences (*p<0.05, ***p<0.001).

In *S. cerevisiae*, it is known that after pheromone recognition, the opposite mating type cell develops projections called *shmoos*, characterized by a polarized growth along a pheromone gradient towards a mating partner [38]. Therefore, the response of AGLPreB cells to synthetic Pbα (WCTRPGQGC) was first monitored by observation of *shmoo* formation. Maximum *shmoo* formation was observed after 5 h for AGLPreB exposed to synthetic Pbα (Figure 4C, D, E, F), with a decrease after 9 h (Figure 4E). A similar pattern was observed for the wild-type *S. cerevisiae MAT* **a** strain BY4741 upon exposure to the cognate synthetic α-pheromone (MFα) (Figure 4A, B, E). We also examined the potential for inter-species signalling between α-pheromones and receptors from *S. cerevisiae* and *Paracoccidioides*. Our data shows that the responses were species-specific since the synthetic Pbα was unable to induce *shmoo* formation through activation of Ste2, while synthetic MFα failed to activate PreB (data not shown). Overall these data demonstrate the effectiveness of the Pbα pheromone in the activation of PreB, and establish the proficiency of *S. cerevisiae* as a heterologous system to study *Paracoccidioides* mating machinery, paving the way for further studies.

As shown previously, expression of *PBα* in *Paracoccidioides* is independent of mating type (Figure 2). However, it could not be discounted that mating type-specific post-transcriptional mechanisms contribute to the production of a fully functional pheromone. Hence, we also investigated if strains of both mating-types could express a functional α-pheromone. For this, Pbα was isolated from the supernatants of *Paracoccidioides* cell cultures of Pb01, Pb03, ATCC60855 and T8B1 as well as various co-cultures. Pbα pheromone extracted from *P. brasiliensis* strains Pb03, T8B1 and ATCC60855 did not trigger *shmoo* formation in strain AGLPreB (data not shown). However, Pbα extracted from *Paracoccidioides* co-cultures Pb01/Pb03 and Pb01/Pb60855 as well as from the Pb01 mono-culture, induced *shmoo* formation in AGLPreB (Figure 4F) in a manner dependent on the expression of *PREB* (not shown). Moreover, Pbα isolated from the supernatant of *S. cerevisiae* cultures overexpressing *PBα* (AGMPbα strain) also induced *shmoo* formation (Figure 4F). The quantification of *shmoo* formation revealed an induction ranging from 1% to 10%, and was significantly higher for pheromone isolated from co-cultures (Figure 4F). Taken together, the data presented suggests that at least *P. lutzii* Pb01 produces an active α-pheromone.

### The *Paracoccidioides* α-pheromone Induces Cell Cycle and Growth Arrest in *S. cerevisiae* Expressing the α-pheromone Receptor

**Figure 5:**
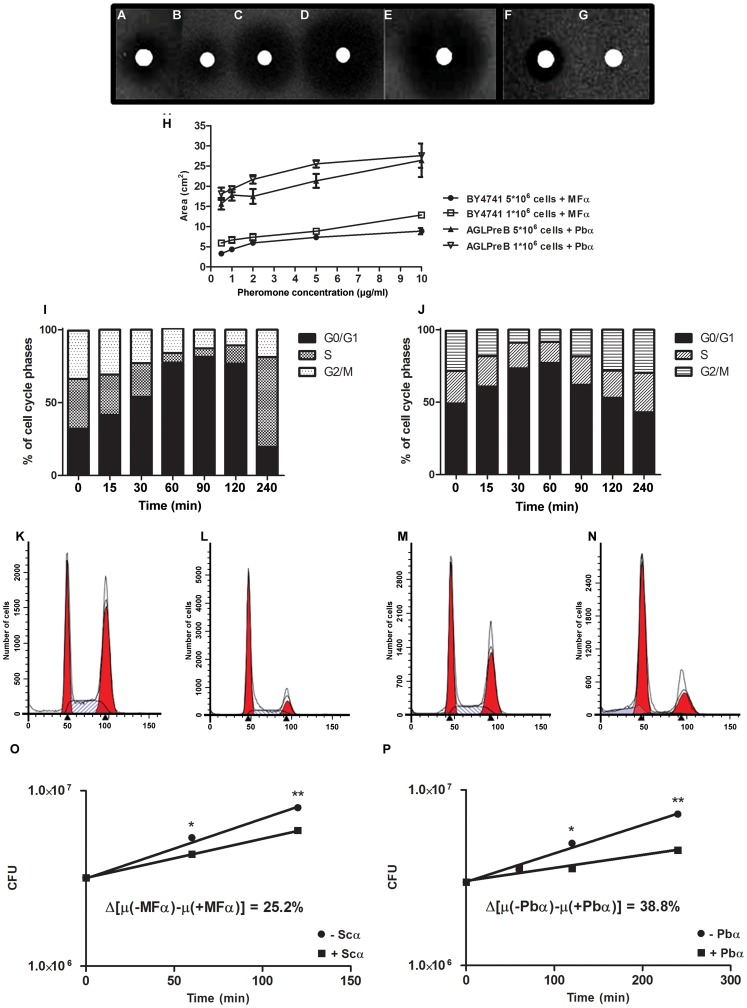
α-pheromone activates PreB receptor, inducing growth and cell cycle arrest in the *S. cerevisiae* model. Halo formation in *S. cerevisiae* AGLPreB stimulated with Pbα pheromone extracted from *P. lutzii* Pb01 monoculture (A); stimulated with 0.8, 4.0 and 40 µg synthetic Pbα pheromone (B–D respectively) or Pbα pheromone extracted from *S. cerevisiae* AGMPbα (E). *S. cerevisiae* BY4741 stimulated with 10 µg synthetic MFα (F) and with 20 µg synthetic Pbα (G). Quantification of growth inhibition zones (cm^2^) upon exposure to α-pheromones. Halos were recorded after 24 hrs of incubation and standard deviations indicated by error bars (H). Cell cycle arrest analysis for *S. cerevisiae* BY4741 upon addition of synthetic MFα (20 µg/ml) (I) and *S. cerevisiae* AGLPreB upon addition of synthetic Pbα pheromone (40 µg/ml) (J). (K–N) FCM profiles of α-pheromone-induced cell cycle arrest in haploid cells showing n and 2 n nuclear DNA content: BY4741 induced with 20 µg synthetic MFα (K–L) and *S. cerevisiae* AGLPreB induced with 40 µg synthetic Pbα pheromone (M–N). Growth rate evaluation of either *S. cerevisiae* BY4741 (O) or AGLPreB (P) upon addition of the cognate synthetic α-pheromone. Error bars are indicated, and asterisks show significant differences (*p<0.05, **p<0.01).

Efficient cell fusion during mating requires cell synchronization, which is achieved by cell cycle arrest in the G1 phase, ultimately resulting in growth arrest [39]. Pheromone-induced growth and cell cycle arrest were assessed in strain AGLPreB exposed to Pbα (Figure 5). Our results show that AGLPreB undergoes growth arrest, with increased halo areas upon exposure to higher amounts of synthetic pheromone (Figure 5B–D, H). Pbα extracted from strain Pb01 cultures (Figure 5A) also induced growth arrest, while this was not the case for *MAT1-1* strain T8B1 or *MAT1-2* strains Pb60855 and Pb03 (data not shown). These results are in line with those obtained for the same set of strains for the induction of shmoo formation. In addition, Pbα isolated from *S. cerevisiae* AGMPbα cultures also resulted in halo formation (Figure 5 E), confirming that *S. cerevisiae* is capable of producing a functional Pbα pheromone.

Flow cytometric analysis of DNA content demonstrated pheromone-induced cell cycle arrest, as indicated by the increase of the G0/G1 DNA peak (n) after the addition of Pbα synthetic pheromone to the AGLPreB strain (Figure 5J, M and N). The maximum cell cycle arrest was achieved 60 min after pheromone stimulation, with an increase of ≈30% of cells arrested in the G1-phase. Growth arrest was also confirmed by the decrease of cell growth rate upon pheromone stimulation (Figure 5P).

Identical results were obtained for wild-type *S. cerevisiae* exposed to its cognate synthetic MFα pheromone (Figure 5F, H, I, K, L and O).

### Heterologous Expression of *Paracoccidioides* α-pheromone and its Receptor PreB Restore the Mating Ability of the Corresponding *S. cerevisiae* Null Mutants

**Figure 6:**
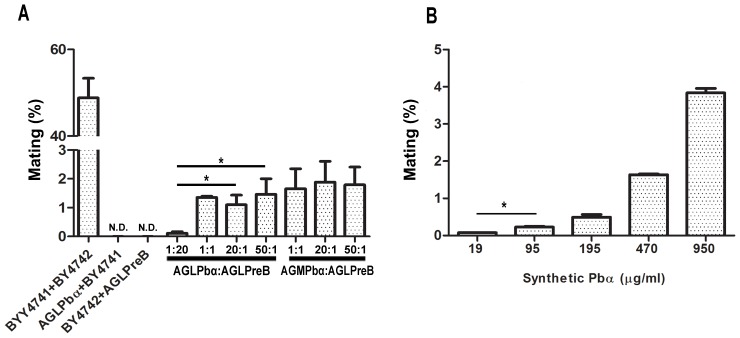
α-pheromone and its cognate receptor functionally complement mating in the corresponding *S. cerevisiae* mutants. Mating efficiency of *S. cerevisiae* null mutants, expressing *Paracoccidioides* α–pheromone (AGLPbα and AGMPbα) and its cognate receptor (AGLPreB), crossed at different ratios after incubation for 5 hrs (A). (B) Mating efficiency after 5 hrs incubation of *S. cerevisiae* strains AGΔScα and AGLPreB, upon the addition of different concentrations of *Paracoccidioides* synthetic α-pheromone. Error bars are indicated, and asterisks show significant differences (*p<0.05).

We have demonstrated that the *PBα* gene from *Paracoccidioides* encodes a functional α-pheromone that signals through its cognate receptor PreB, using *S. cerevisiae* as a host model. To evaluate the proficiency of the heterologous mating system we investigated the ability of the *Paracoccidioides* pheromone-receptor system to rescue mating of cognate *S. cerevisiae* mutants. Thus, a *S. cerevisiae* double null mutant for the MFα(1/2) genes expressing the *PBα* gene (using low and medium-copy number vectors in strains AGLPbα and AGMPbα, respectively) was crossed with the *PREB*-expressing strain AGLPreB. Resulting diploid cells were scored by the acquisition of both methionine and lysine prototrophy. Mating efficiency was higher for crosses between AGLPreB and AGMPbα (medium-copy plasmid) compared to crosses using AGLPbα (low-copy plasmid) (Figure 6A). Mating efficiencies were, in general, relatively low for the heterologous mating pairs (below 10%), similar to those described for other heterologous systems expressed in *S. cerevisiae* [40], compared with 50% diploids observed for the control system (*S. cerevisiae* wild-type strains). These results, together with those obtained with mixtures of AGLPbα and AGLPreB at different cell number ratios, suggested that α-pheromone was a limiting factor for mating (Figure 6A). To assess this, we evaluated mating following the addition of exogenous synthetic pheromone at different concentrations to the BY4742 and AGLPreB mating cross. The results obtained show a dose response effect, although the mating efficiencies are still low (Figure 6B). A similar assay for the heterologously expressed Ste2 homolog from *C. albicans* previously showed diploid formation of up to 80% [41], although it has to be noted that the *C. albicans* homolog CaSte2 is more similar to Ste2 than PreB (36 vs 28% identity).

## Discussion

In this work, we investigated the functionality of the sexual reproduction machinery of the genus *Paracoccidioides*. We demonstrate that the genomes of these fungi encode a functional α-pheromone that is able to activate its cognate receptor PreB, but we also show that *P. brasiliensis* ATCC60855 and Pb03, both *MAT1-2* strains, as well as the *MAT1-1* strain *P. lutzii* Pb01 are apparently insensitive to α-pheromone. This suggests that pheromone signalling is somehow blocked in these *Paracoccidioides* strains, probably due to low transcription levels of the upper components of the signalling pathway.

Notably, we could demonstrate that *P. lutzii* Pb01 expresses a functional α-pheromone. The identified mature pheromone sequence was shown to be identical to the one described in *H. capsulatum,* although the precursor was slightly different. In both *Paracoccidioides* and *Histoplasma* the precursor peptide encodes only one copy of the mature pheromone, which is in contrast to the majority of ascomycetes that encode two or more copies of the mature pheromone in the precursor [34].

The predicted α-pheromone and its cognate receptor were individually overexpressed in *S. cerevisiae* null mutants of the homologous genes (constructed in strains of opposite mating types), and the functionality of this signaling pair was confirmed by their ability both to restore the hallmarks of pheromone signaling (shmoo induction and cell cycle and growth arrest) and to complement (at least partially) the mating deficiency of the host. Moreover, we investigated the specificity of the α-pheromone–receptor interaction that regulates the pheromone-sensing pathway. We demonstrate that there is considerable specificity in the interaction of Ste2 and PreB receptors with their cognate pheromone ligands. Thus, incubation of BY4741 and AGLPreB with Pbα and MFα synthetic pheromones respectively did not show evident *shmoo* formation, cell cycle and growth arrest, contrarily to what was observed when stimulation was performed with the corresponding pheromone. The observed pheromone specificity can be attributed to the differences in protein sequences and topology of Ste2 and PreB. In particular the N-terminal region and trans-membrane domain 1, which have been implicated in pheromone binding [42], show low similarity between these receptors (Figure S2).

Functioning of the *Paracoccidioides* α-pheromone/α-pheromone receptor pair in the heterologous host was evident, although the observed mating efficiencies were low. Similar low mating efficiencies of heterologously expressed mating genes in *S. cerevisiae* models have been shown in other studies [40], [41]. Interestingly, also expression of endogenous pheromones and pheromone receptors leads to reduced mating efficiencies that are most probably related to the expression system used [40]. In addition, the low identity between PreB and Ste2 (28%) might cause a less efficient interaction of the PreB receptor with the pheromone-response pathway G-protein of the host. In fact, topology predictions for these α-pheromone receptors suggested that the third intracellular loop, important for the interaction of Ste2 with the G-protein, is different. Nevertheless, the known essential markers in the third intracellular loop needed for the activation of the intracellular mating pathway in *S. cerevisiae* are shared between Ste2 and PreB. In particular, the two essential amino acids R233 and F241 in Ste2 [43] are also present and separated by the same number of amino acids in PreB (R223 and F231), and the triplet LGL is located at the same position in between these residues for both receptors (Figure S2). The topology prediction however places phenylalanine residue F231 into the transmembrane domain of PreB. Furthermore, the second loop in PreB is much longer than that of Ste2, which can also contribute to a general structural alteration of the intracellular receptor domain that culminates in a reduced signaling efficiency. Accordingly, *PbGPA1* was unable to rescue the mating ability of the corresponding *S. cerevisiae GPA1* null mutant. However, mating was rescued by simultaneous expression of both *PREB* and *PbGPA1* in a *S. cerevisiae* double mutant (Figure S3).

In the heterologous mating experiments we could show that α-pheromone was a limiting factor because higher concentration of pheromone resulted in increased mating efficiency. Even so, with the exogenous addition of the synthetic Pbα pheromone at a very high concentration, a mating efficiency of only 3.7% was achieved. It is likely that either the binding affinity of the ligand for the receptor or the interaction of the receptor with the signal transduction pathway, or both, contribute to the reduced pheromone bioactivity. Further studies are therefore needed, eventually using pheromone analogs, to evaluate their ability to activate the signaling machinery.

One of the most revealing aspects of the current work is the demonstration of the biochemical proficiency of the pheromone-signaling machinery of *Paracoccidioides* genus, at the level of α-pheromone and its receptor, while at the same time signaling seems to be impaired. In fact, our results also show that mating gene expression in *Paracoccidioides* species is very low, especially the upper components of the signalling cascade (*PREB*, *GPA1*, *STE4* and *STE18*). Moreover, expression of mating-related genes was not inducible through stimulation with synthetic *Paracoccidioides* α-pheromone. This could suggest that due to the low basal gene expression the number of PreB receptor molecules present on the cell surface is too low to elicit a mating response, as well as the other components of the signaling pathway. Therefore, further research is underway to identify the environmental conditions that stimulate mating gene expression, as well as to detect low frequency mating events in this fungal pathogen via genetic approaches with dominant-marked strains. The confirmation of a sexual cycle in *Paracoccidioides* and the molecular characterization of its mating system are of major importance, since it will provide essential knowledge on basic biological and evolutionary aspects of these fungi. In addition, identification of sexual mechanisms will support genetic studies in this fungus and this information will contribute valuable new insights into the long-standing problem of sexuality/asexuality, which has not been fully elucidated in many fungi.

## Supporting Information

Figure S1 **Alignment of α-pheromone cDNA sequences of** ***P. lutzii*** **Pb01 and** ***P. brasiliensis*** **ATCC60855 and partial genomic DNA sequence (minus strand) of the predicted ORF PAAG_00855 from** ***P. lutzii*** **Pb01.** The location of the stop codon of PAAG_00855 is indicated by a red box. Nucleotides in the minus strand DNA sequence of PAAG_00855 obeying the canonical mRNA splicing motifs are underlined. Nucleotide translation to pheromone precursor is shown below the DNA sequence. Splicing of the 79 bp intron results in the proline (P) codon CCC detected in the cDNA (boxed positions).
(TIF)

Figure S2 **Topology of α-pheromone receptor Ste2 from** ***S. cerevisiae*** **(A), PreB from** ***P. lutzii*** **Pb01 (B), and PreB from** ***H. capsulatum*** **(C).** Topology predictions were made using TMHMM Software and visualization done using the TMRPres2D tool. Essential residues for Gpa1 interaction of Ste2 in the third intracellular loop (IL3) are highlighted in red. Regions and residues involved in pheromone recognition and specificity of Ste2 are highlighted in blue. Corresponding regions and putative residues in PreB are indicated in similar colors.
(TIF)

Figure S3 **Mating efficiencies of** ***S. cerevisiae*** **null mutants heterologously expressing the** ***Paracoccidioides*** **α-pheromone receptor PreB and G-protein PbGpa1.** Strains and plasmids used in mating assays (A). Mating efficiencies of mate pairs (B). Mating assays were performed as described in the Materials and Methods. PbGpa1 on its own is not able to restore mating in a BY4741_GPA1 strain, but the *Paracoccidioides* α-pheromone receptor PreB and G-protein PbGpa1 collectively restore the mating ability in the corresponding *S. cerevisiae* double mutant.
(PDF)

Table S1 **Primers used in this study.** Sequences for homologous recombination (HR) in *S. cerevisiae* are indicated by shading. Start and stop codons are underlined.
(PDF)
