# Supplementary figures and images for: Functionality of the Paracoccidioides Mating α-Pheromone-Receptor System
Source: PLoS One. 2012 Oct 4;7(10):e47033. doi: 10.1371/journal.pone.0047033 (PMC3464258; doi:10.1371/journal.pone.0047033)

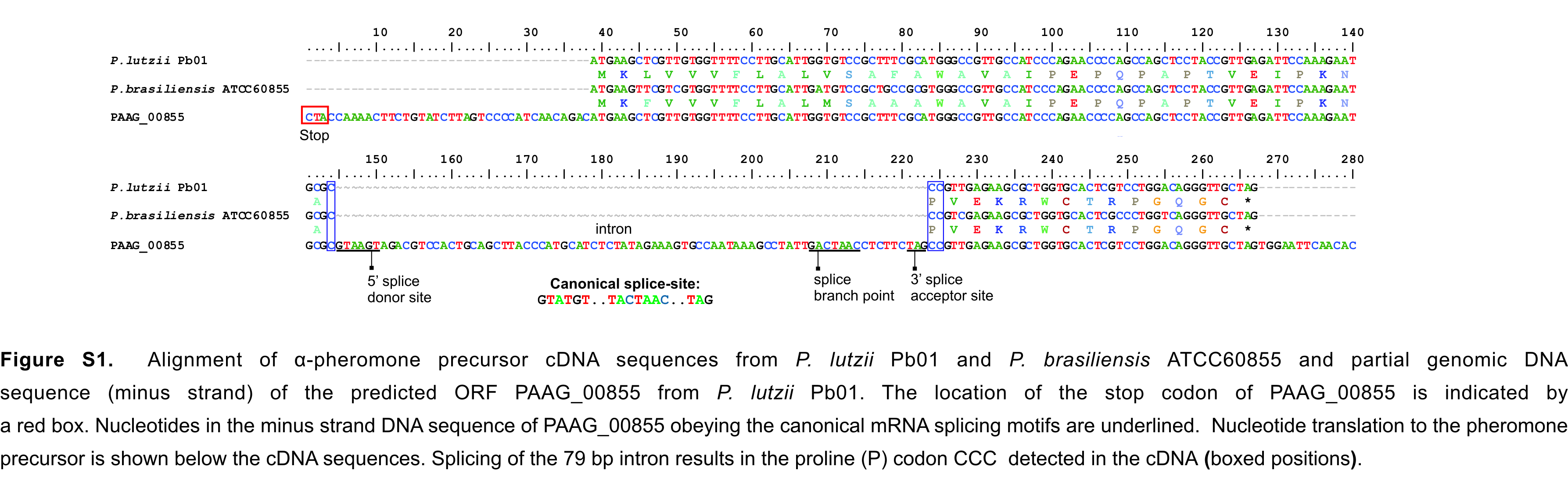

Supplement: Figure S1 — Alignment of α-pheromone cDNA sequences of P. lutzii Pb01 and P. brasiliensis ATCC60855 and partial genomic DNA sequence (minus strand) of the predicted ORF PAAG_00855 from P. lutzii Pb01. The location of the stop codon of PAAG_00855 is indicated by a red box. Nucleotides in the minus strand DNA sequence of PAAG_00855 obeying the canonical mRNA splicing motifs are underlined. Nucleotide translation to pheromone precursor is shown below the DNA sequence. Splicing of the 79 bp intron results in the proline (P) codon CCC detected in the cDNA (boxed positions). (TIF) [file pone.0047033.s001.tif]

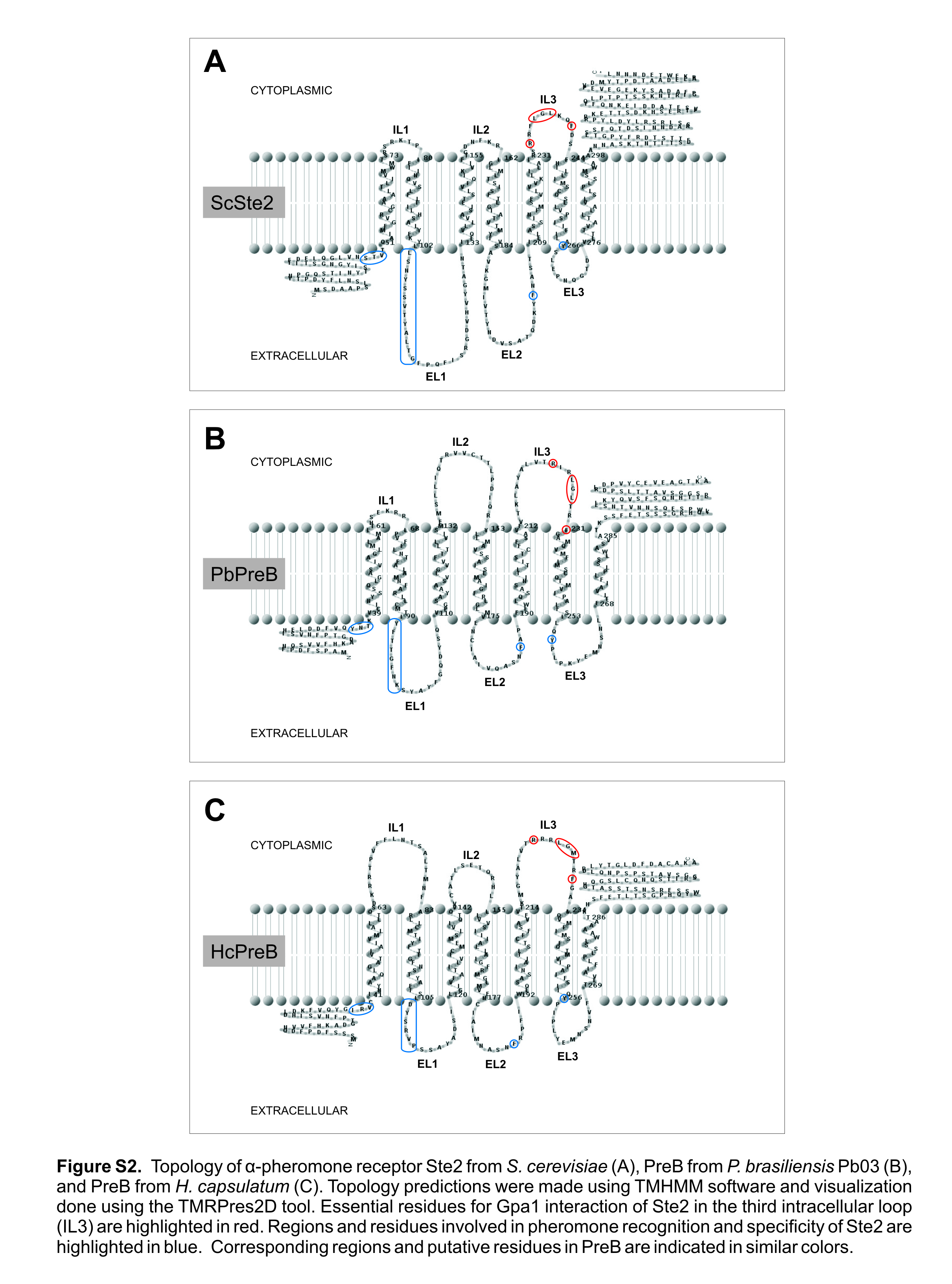

Supplement: Figure S2 — Topology of α-pheromone receptor Ste2 from S. cerevisiae (A), PreB from P. lutzii Pb01 (B), and PreB from H. capsulatum (C). Topology predictions were made using TMHMM Software and visualization done using the TMRPres2D tool. Essential residues for Gpa1 interaction of Ste2 in the third intracellular loop (IL3) are highlighted in red. Regions and residues involved in pheromone recognition and specificity of Ste2 are highlighted in blue. Corresponding regions and putative residues in PreB are indicated in similar colors. (TIF) [file pone.0047033.s002.tif]
