# Supplementary material for: Functionality of the Paracoccidioides Mating α-Pheromone-Receptor System
Source: PLoS One. 2012 Oct 4;7(10):e47033. doi: 10.1371/journal.pone.0047033 (PMC3464258; doi:10.1371/journal.pone.0047033)
Supplement: Figure S3 — Mating efficiencies of S. cerevisiae null mutants heterologously expressing the Paracoccidioides α-pheromone receptor PreB and G-protein PbGpa1. Strains and plasmids used in mating assays (A). Mating efficiencies of mate pairs (B). Mating assays were performed as described in the Materials and Methods. PbGpa1 on its own is not able to restore mating in a BY4741_GPA1 strain, but the Paracoccidioides α-pheromone receptor PreB and G-protein PbGpa1 collectively restore the mating ability in the corresponding S. cerevisiae double mutant. (PDF) [file pone.0047033.s003.pdf]

(A)

Strains/Plasmids	Genotype	Source
Strains		
BY4741 *ΔGPA1*	*MAT***a** *GPA1*::KanMX4	EUROSCARF
AG*ΔGPA1ΔSTE2*	*MAT***a** *GPA1*::hph; *STE2*::kanMX4	This study
AGLPbGpa1	*MAT***a** *GPA1*::hph pLPbGpa1	This study
AGLPbGpa1PreB	*MAT***a** *GPA1*::hph pLPbGpa1; *STE2*::kanMX4 pLPreB	This Study
Plasmids		
pLPbGpa1	pCM189::PbGPA1 Ampicilin^R/Histidine$^+$	This study

(B)

Mate pair[a]	% Mating[b]
BY4741 X BY4742	48.8 ± 4.53
BY4741 X AGLPbα	Not detected
AGLPreB X BY4742	Not detected
AGLPreB X AGLPbα	2.53 ± 0.87
AGLPbGpa1 X BY4742	Not detected
AGLPreBPbGpa1 X AGLPbα	2.92 ± 0.56

[a] Strains as described in (A)
[b] Percentage mating ± standard deviation

Figure S3: Mating efficiencies of *S. cerevisiae* null mutants heterologously expressing the *Paracoccidioides* α-pheromone receptor PreB and G-protein PbGpa1. Strains and plasmids used in mating assays (A). Mating efficiencies of mate pairs (B). Mating -assays were performed as described in the Materials and Methods. PbGpa1 on its own is not able to restore mating in a BY4741_*GPA1* strain, but the *Paracoccidioides* α-pheromone receptor PreB and G-protein PbGpa1 collectively restore the mating ability in the corresponding *S. cerevisiae* double mutant.
