# Supplementary material for: Functionality of the Paracoccidioides Mating α-Pheromone-Receptor System
Source: PLoS One. 2012 Oct 4;7(10):e47033. doi: 10.1371/journal.pone.0047033 (PMC3464258; doi:10.1371/journal.pone.0047033)
Supplement: Table S1 — Primers used in this study. Sequences for homologous recombination (HR) in S. cerevisiae are indicated by shading. Start and stop codons are underlined. (PDF) [file pone.0047033.s004.pdf]

Table S1. Primers used in this study. Sequences for homologous recombination (HR) in *S. cerevisiae* are indicated by shading. Start and stop codons are underlined.

Primer	Sequence 5´-3´	Target	Reference
Homologous recombination			
PREB-HR-Fw	CACTAAATTACCGGATCAATTCGGGATGGCACCCTCATTCGACCCCTTC	pCM189 / *PREB*	This study
PREB-HR-Rev	TCGATGTTAACAGGCCTGTTTAAACTCAGGCCTTTGTGCCAGCTTC	pCM189 / *PREB*	This study
Pbα-HR-Fw	CACTAAATTACCGGATCAATTCGGGATGAAGTTCGTCGTGGTTTTCCTTGC	pCM189-pCM190 / *PBα*	This study
Pbα-HR-Rev	TCGATGTTAACAGGCCTGTTTAAACCTAGCAACCCTGACCAGGGCGAGTG	pCM189-pCM190 / *PBα*	This study
MFα2-HR-HPH-Fw	GCTAGTGTTCACTTGCTCATTGATGTCCCGAGAAAGGAAGGGAAGAAAGCGAAAGGAG	*MFα2* flank / HPH	This study
MFα2-HR-HPH-Rev	GGCCAATTATTACTGCTAAAGATAAACTCCACAGGAAACAGCTATGACCATGATTAC	*MFα2* flank / HPH	This study
Gene replacement confirmation			
pCM-Conf-Fw	GCATGCATGTGCTCTGTATG	pCM189 / pCM190	This study
pCM-Conf-Rev	TTTCGGTTAGAGCGGATGTG	pCM189 / pCM190	This study
HPH-Conf-Fw	CGCAAGGAATCGGTCAATAC	HPH	This study
HPH-Conf-Rev	AAAGCATCAGCTCATCGAGA	HPH	This study
MFα-Conf-Rev	GTCCGAAAAATTGAAAGTC	*MFα2* flank	This study
Real-time PCR			
MAT11-RT-Fw	CATTCAGAAGCTTCTACTCTAC	*MAT1-1*	This study
MAT11-RT-Rev	CCTTCGCAAGGATTGCCCAC	*MAT1-1*	This study
MAT12-RT-Fw	AACGACATATCGATACTCCTTG	*MAT1-2*	This study
MAT12-RT-Rev	GATAGTAAGGGTGATCTTTG	*MAT1-2*	This study
Pbα-RT-Fw	GTCGTGGTTTTCCTTGCATTG	*Pbα*	This study
Pbα-RT-Rev	TCTCGACGGGCGCATTC	*Pbα*	This study
PREB-RT-Fw	GTGATCCCAGCCATCTTCTC	*PREB*	This study
PREB-RT-Rev	GTAGCCACTGAAGCCCATAG	*PREB*	This study
PREA-RT-Fw	TCCCCAAGAAACATCAGTCC	*PREA*	This study
PREA-RT-Rev	CATGACCATGCTAGAGGGATG	*PREA*	This study

Primer	Sequence 5´-3´	Target	Reference
Real-time PCR			
GPA1-RT-Fw	ACTGAAGAGGGATAAGATG	*GPA1*	This study
GPA1-RT-Rev	CATAGCCTCGAGAATAAC	*GPA1*	This study
STE4-RT-Fw	CTGGGAAATCAGTACAAAC	*STE4*	This study
STE4-RT-Rev	ACCTTCTCTCCTCTTAGAAC	*STE4*	This study
STE18-RT-F1	CTATCCCACACCATTCTAG	*STE18*	This study
STE18-RT-R1	GTACGTTGAGCTCATTTAG	*STE18*	This study
STE20-RT-F1	TGGATATGATAACGAGAC	*STE20*	This study
STE20-RT-R1	TAATATCCACCATAGTCTGC	*STE20*	This study
STE11-RT-F1	CAGCTCACAAATCATCTAC	*STE11*	This study
STE11-RT-R1	CGTATACTATGTAGCCAATC	*STE11*	This study
STE50-RT-F1	GCCAAGAACTATCATCAC	*STE50*	This study
STE50-RT-R1	ACTCTTTAGAATCGTCAGTC	*STE50*	This study
STE7-RT-F1	GCCTATTCTTGATGACTTTG	*STE7*	This study
STE7-RT-R1	GTAGGATTTGCGGTTGTG	*STE7*	This study
KSS1-RT-F1	GACAACTCTGGATTTATGAC	*KSS1*	This study
KSS1-RT-R1	GATAAGGGTAAGTTGGTG	*KSS1*	This study
STE12-RT-Fw	AGAAACCTTCATGCTCTC	*STE12*	This study
STE12-RT-Rev	CAACTGGTGCAGATATAAG	*STE12*	This study
Tub2F	AGCCTTGCGTCGGAACATAG	*β-tubulin (TUB2)*	Marques *et al.*, 2004
Tub2R	ACCTCCATCCAGGAACTCTTCA	*β-tubulin (TUB2)*	Marques *et al.*, 2004
